# Anti-PD-1 Immunotherapy Combined With Stereotactic Body Radiation Therapy and GM-CSF as Salvage Therapy in a PD-L1-Positive Patient With Refractory Metastatic Thyroid Hürthle Cell Carcinoma: A Case Report and Literature Review

**DOI:** 10.3389/fonc.2021.782646

**Published:** 2021-11-23

**Authors:** Haihua He, Tangpeng Xu, Ping Li, Guohua Jia, Xiangpan Li, Qibin Song

**Affiliations:** ^1^ Cancer Center, Renmin Hospital of Wuhan University, Wuhan, China; ^2^ Department of Oncology, Renmin Hospital of Wuhan University, Wuhan, China

**Keywords:** thyroid Hürthle cell carcinoma, immunotherapy, radiotherapy, PD-1/L1, GM-CSF

## Abstract

Thyroid Hürthle cell carcinoma, known as thyroid eosinophilic carcinoma, is a rare pathological type of differentiated thyroid cancer (DTC), representing 3-4% of all thyroid cancers. However, given the high risk of invasion and metastasis, thyroid Hürthle cell carcinoma has a relatively poor prognosis. Traditional treatment methods have limited effects on patients with metastatic thyroid cancers. Developing a valuable therapy for advanced thyroid carcinomas is an unfilled need, and immunotherapy could represent another choice for these tumors. We herein reported the case of a patient with recurrent advanced thyroid Hürthle cell cancer and positive programmed death-ligand 1 (PD-L1) expression, who suffered tumor progression after re-surgery, radiotherapy, and targeted therapy. It is encouraging that PD-1 inhibitors in combination with GM-CSF and stereotactic body irradiation (SBRT) on metastatic disease have a significant anti-tumor effect.

## Introduction

Thyroid cancer is the most common endocrine malignancy, with an increasing global incidence in recent decades. It is mainly divided into four histological types, namely papillary thyroid carcinoma (PTC), follicular thyroid carcinoma (FTC), medullary thyroid carcinoma, and thyroid anaplastic carcinoma. The PTC and FTC are collectively referred to as differentiated thyroid cancer (DTC) (1, 2). Hürthle cell carcinoma (HCC) has been traditionally classified as a variant of follicular cancer (3). However, in the 4^th^ edition of the WHO Classification, Non-invasive cases are called Hürthle cell adenoma, and cases with capsular or vascular invasion are called Hürthle cell carcinoma, both of which are composed of more than 75% oncocytic cells (1, 4).

Therapies for thyroid cancer mainly include surgery, radioactive iodine (RAI), and TSH Inhibition. Thyroid cancer is generally associated with a good prognosis, with a 5-year overall survival (OS) rate of 98%. Yet, roughly 10% of patients with DTC experience advanced invasive primary disease, 5% have distant metastases, and 20–30% experience disease relapse (5). Age, tumor size, and gender are prognostic factors of HCC, and tumor extension and recurrence are often associated with poor prognosis and increased mortality (6). Treatment protocols for recurrent thyroid cancer are finite. Sorafenib and lenvatinib are oral multikinase inhibitors targeting VEGFR and are approved for radioiodine(RAI)-refractory differentiated thyroid cancer. Besides, immunotherapy targeting the PD-1/PD-L1 axis inhibited tumor growth in several pre-clinical and clinical trials of aggressive thyroid cancers (7). The expected overall response rate of TC patients to immune checkpoint inhibitors is 20-40%, which is similar to other cancers (8).

**Figure 1:**
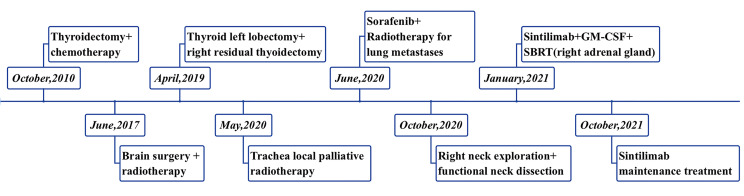
Timeline of the case history.

The clinical use of immune checkpoint inhibitors has extended to a variety of malignancies, but are not yet approved for advanced thyroid carcinoma (9). We can see their indications in preclinical studies, a few case reports, and clinical trials (9). There is no standard treatment to improve immune response and correct drug resistance, the most important and effective measure is immunotherapy combined with other therapies, aiming to turn ‘cold’ tumors into ‘hot’ tumors (10). This case report demonstrated the effectiveness of immunotherapy combined with SBRT and GM-CSF for HCC (**Figure 1**), but the role of the combination therapy needs further study.

## Case Presentation

A 35-year-old man visited the clinic with a neck mass on October 20, 2010. Ultrasonography showed an abnormal mass in the right lobe of the thyroid gland. Thereafter, the patient underwent partial right thyroidectomy and the pathological examination revealed a thyroid follicular adenoma with hemorrhagic cystic degeneration. However, the pathology consultation in another hospital indicated Hürthle cell carcinoma (eosinophilic follicular carcinoma, poorly differentiated) and the immunohistochemistry results(**Supplementary Figure 1**) showed TTF-1 (+++), CK8/18 (++), PCK (+), VIM (+), TG (+), SYN (-), and CGA (-). Magnetic resonance imaging (MRI) and computed tomography (CT) one month later indicated a right inferior thyroid space-occupying lesion and multiple nodules in both lungs and the liver. Subsequently, the patient received eight cycles of chemotherapy consisting of docetaxel and carboplatin. About seven years later, on June 23, 2017, the patient underwent brain surgery and postoperative radiotherapy (45 Gy in 10 fractions) for a metastatic tumor in the left temporal lobe. Thereafter, the patient had a follow-up of about 1.5 years, with no clinical or radiographic evidence of recurrence and metastasis. On April 29, 2019, due to the enlargement of the neck mass, the patient underwent thyroid lobectomy on the left, resection of residual tissue on the right, bilateral recurrent laryngeal nerve exploration, and central lymph node dissection.

**Figure 2:**
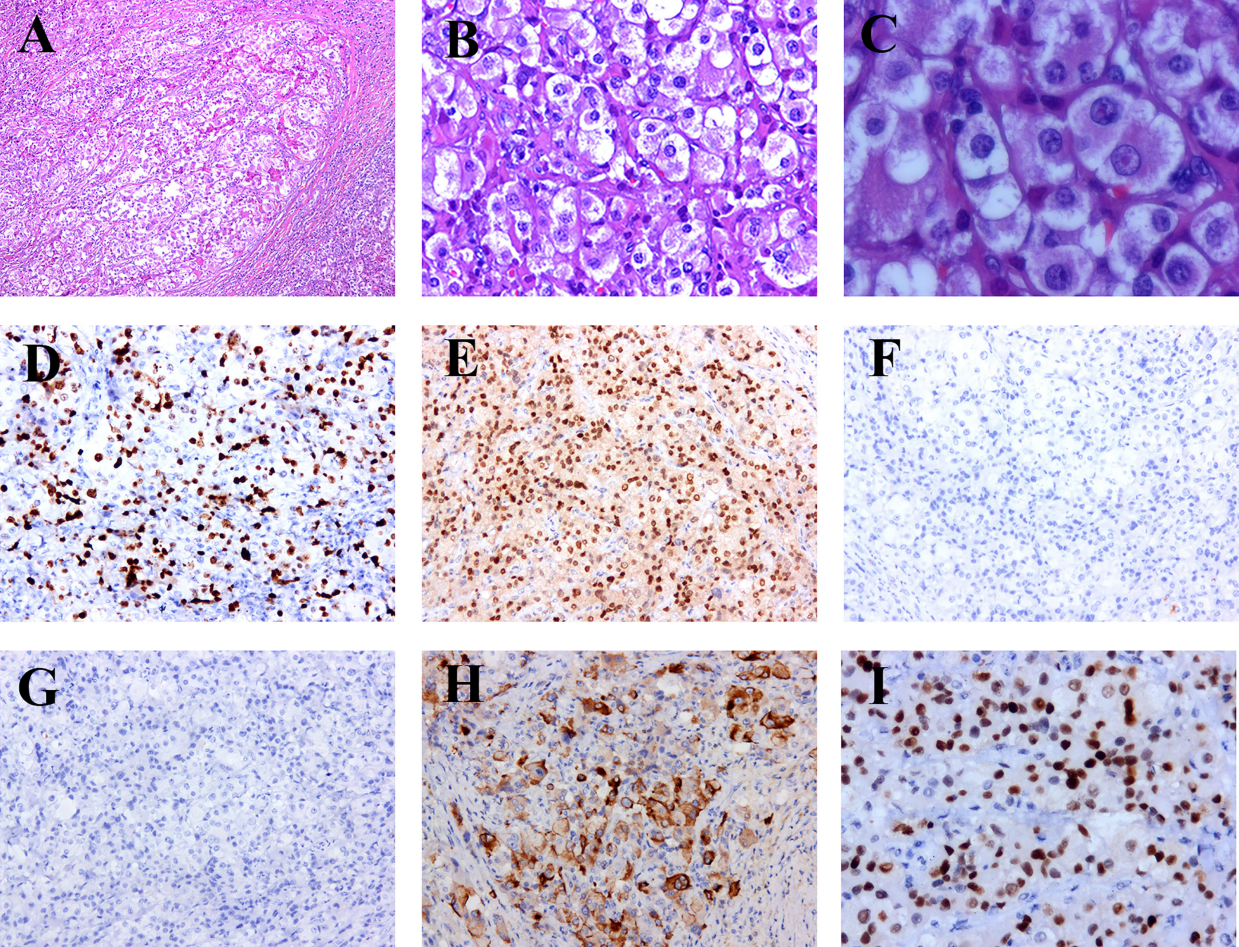
Hisopathological and immunohistochemical examinations. **(A)** 100 times. Dense eosinophilic cytoplasm, with red staining and large obvious nucleous. **(B)** 200 times. Large cell with abundant eosinophilic cytoplasm and a large hyperchromatic nucleus with a prominent nucleolus, obvious nuclear heteromorphism, and visible mitotic strutures. **(C)** 400 times. **(D)** The positive rate of Ki67 is 70%. **(E)** TTF-I positive. **(F)** CK19 negative. **(G)** TG negative. **(H)** PCK focal positive. **(I)** P53 part positive.

On April 10, 2020, the patient visited our hospital due to hemoptysis and bronchoscopy showed new cauliflower-like growth in the upper trachea about 2 cm from the glottis. The pathological examination indicated thyroid Hürthle cell carcinoma. The CT scan indicated multiple enhanced nodules in the endotracheal cavity, IV region of the right neck, and both lungs. These results indicated the recurrence and metastasis of thyroid cancer. To relieve the symptoms of bleeding and compression, oncologists administered tracheal local palliative radiotherapy (70 Gy in 35 fractions). Genetic tests were performed on June 8, 2020, and the heterozygous mutation c.2307G > T (p. Leu 769) was detected in the RET gene, while no mutation was detected in the BRAF gene, RAS (KRAS/NRAS) gene, PIK3CA gene and TP53 gene (wild-type). The patient was started on Sorafenib (400 mg, twice a day) as targeted therapy. One month later, a CT scan showed that the lung nodules were larger than before, and the patient was treated with radiotherapy (48 Gy in 6 fractions) for pulmonary metastases. After two cycles of targeted therapy, re-examination revealed that the original neck and lung lesions were smaller, but the soft tissue masses from the front lower neck to the front chest wall on the right side had enlarged. The multi-disciplinary team (MDT) suggested right neck exploration plus functional neck dissection. The postoperative pathology (**Figure 2**) indicated metastasis of thyroid Hürthle cell cancer, and the immunohistochemistry showed CK19 (-), P53 (+), PCK (+), Tg (-), TTF-1 (+) and Ki67 (+, 70%).

**Figure 3:**
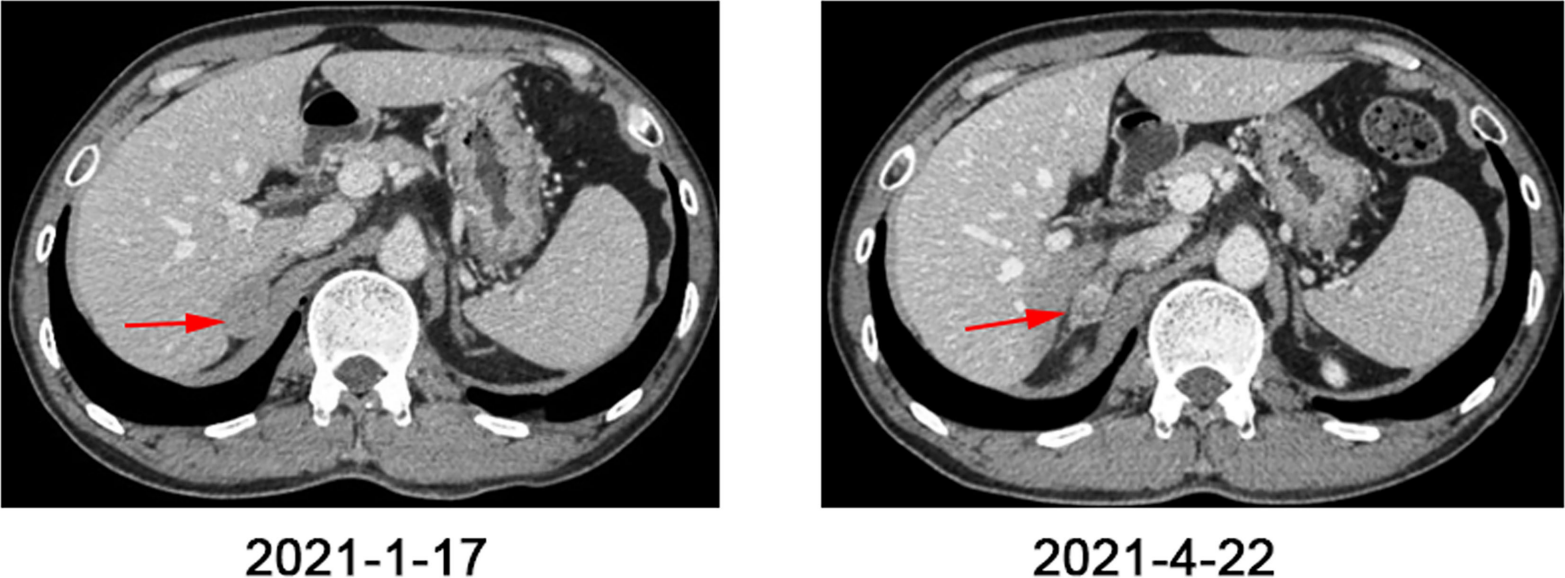
Abdomen CT scans before and after one cycle of triple-combination therapy and two cycles of sintilimab treatment.

**Figure 4:**
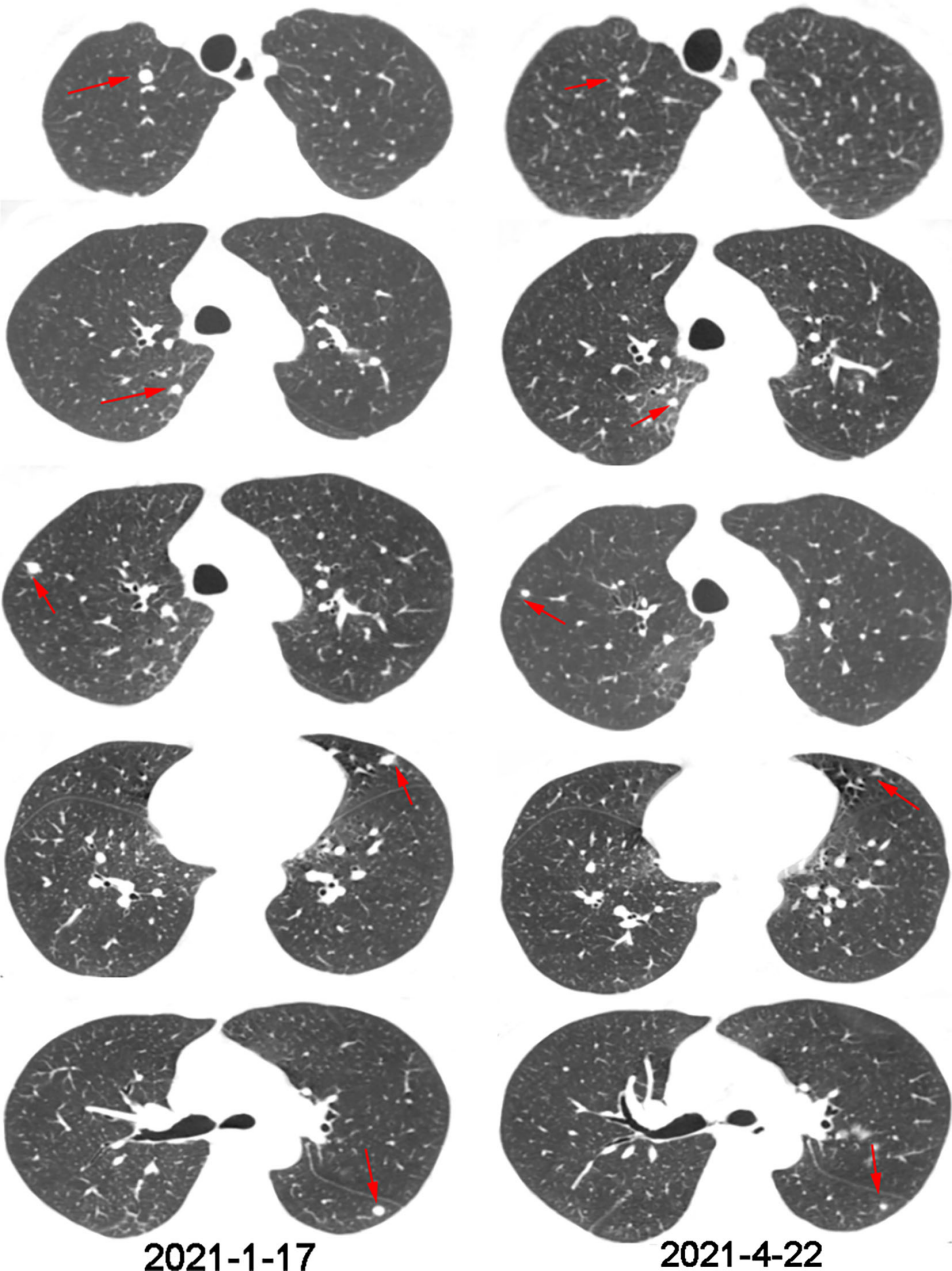
Chest CT scans before and after one cycle of triple-combination therapy and two cycles of sintilimab treatment.

Three months later, a CT scan showed more and larger nodules in both lungs, and newly enlarged nodules in the right adrenal gland (**Figures 3**, **4**). The supplementary immuno-histochemical staining of the tumor tissue showed the PD-L1 expression is positive and the tumor proportion score is 80%(clone 22C3) (**Supplementary Figure 2**). In this subsequent visit, the tumor had progressed, and the MDT recommended triple therapy. On January 25, 2021, the patient received an intravenous PD-1 inhibitor (sintilimab, 200 mg) on the first day, followed by SBRT (32 Gy in 4 fractions) for the metastatic lesion in the right adrenal gland. After SBRT, GM-CSF was injected subcutaneously at a dose of 200 μg per day for 2 weeks. One week after the last dose, the patient had significant improvement in cough and dyspnea. Re-staging scans after two cycles of immunotherapy (three weeks per cycle) revealed a partial response (RECIST v1.1). The CT scans showed that the multiple nodules in the lungs and the right adrenal gland were smaller than before (**Figures 3**, **4**). Moreover, no general adverse events were observed during the treatment. Though the chest CT showed lung inflammation (**Supplementary Figure 3**), the patient had no symptoms. The immunotherapy has been well-tolerated to date.

## Discussion

Hürthle cell carcinoma of the thyroid (HCC), is a type of oncocytic or oxyphilic malignancy comprising of at least 75% Hürthle cells, and with capsular and/or vascular invasion (11). Compared to other types of DTCs, HCC has a higher risk of lymph node metastases and distant metastases and is less sensitive to radioiodine therapy. The 5-year mortality rate of HCC with distant metastases is up to 80% (12). The overall recurrence rate is 12-33%, and the meantime to recurrence is 90.74 months (13). A population-based study on thyroid cancer patients based on the data acquired from the SEER database between 1988 and 2009, analyzed 3311 patients with HCC and 59,585 patients with other types of DTCs (ODTCs). Compared with ODTC, patients with HCC were older (average age, 57.6 years *vs* 48.9 years; p < 0.001) and more likely to be male (31.1% *vs.* 23.0%; p < 0.001). The overall survival rate and disease-specific survival rate of HCC patients were both low (p < 0.001) (14). Vera Wenter et al. conducted a retrospective study in which 126 patients with oncocytic follicular Hürthle cell carcinoma (OFTC) and 252 patients with classical follicular thyroid carcinoma (FTC) were observed, OFTC patients had a significantly higher recurrence rate (17% *vs.* 8%; p = 0.037), while the mean disease-free survival (mDFS) was 20.1 years and 17.9 years separately (p = 0.027). Multivariate analysis showed OFTC (HR 0.502; 95% CI: 0.309–0.816) was the single independent prognostic factor for DFS (15).

Surgical resection is the standard treatment for most patients who suffer from thyroid cancers, while radioactive iodine therapy and TSH suppression treatment are required for those with high-risk factors (7). The therapies targeting MAPK pathway, PI3K/Akt-mTOR pathways, or VEGF, and immunotherapies such as CTLA-4 inhibitor and PD-1/PD-L1 inhibitor, have altered the therapeutic strategies for the aggressive types of thyroid cancer (16). However, due to the rarity of HCC, its pathological features and behavior are not yet clear, and there is currently no consensus regarding the optimal treatment modality. Immune checkpoint inhibitors have demonstrated remarkable efficacy in recent years in the treatment of advanced thyroid carcinoma. In the phase Ib KEYNOTE-028 clinical trial, the safety, tolerability, and anti-tumor activity of pembrolizumab(an anti-PD-1 antibody) were assessed in 22 patients with locally advanced or metastatic DTC and PD-L1 expression positive. Two patients with PTC achieved partial response after 4 and 5 months of treatment, and the response duration time was 20 and 8 months, separately. Moreover, stable disease (SD) was seen in 4 (57%) of 7 patients with follicular tumors and 9 (60%) of 15 patients with papillary tumors. The median progression-free survival (PFS) was 7 months (95% CI: 2-14 months) and the median overall survival (OS) was not reached (17).

Immunotherapy is one of the most successful cancer therapeutic strategies. This treatment approach aims to enhance the immune response of the host to tumors. Although dramatic tumor regression was seen in patients’ responses to ICIs, the majority of the patients show poor responses (18). Current evidence suggests that the efficacy of immunotherapy can be improved by combining other treatments, but the mechanism that drives effective tumor response has not been well elucidated. In NSCLC, a meta-analysis showed that immunotherapy combined with other treatments (e.g. chemotherapy, targeted therapy, double agent immunotherapy) significantly improved OS (HR = 0.74, 95% CI: 0.63–0.85, p = 0.001), PFS (HR = 0.74, 95% CI: 0.63–0.85, p = 0.001), and ORR (OR = 1.51, 95% CI: 1.02–2.00, p < 0.001) (19).

Traditionally, radiation therapy kills tumor cells mainly through direct cytotoxic effects including irreparable DNA damage induced by double-strand breaks, resulting in cell-cycle arrest and cell death (20). Accumulating evidence showed radiotherapy has immunomodulatory effects. Radiotherapy can induce immunogenic cell death and release tumor-associated antigens (TAAs), danger signals, and cytokines (21). Radiotherapy modulates the tumor immune microenvironment, especially the blood vessels, infiltration of lymphocytes, and expression of immune checkpoint ligands (22). Radiation activates dendritic cells and enhances tumor antigen cross-presentation, and increases tumor cell susceptibility to immune-mediated cell death when combined with checkpoint inhibitor immunotherapy (23). Additionally, radiotherapy combined with immune checkpoint blockers (ICBs) enhances both the local impact of radiation and the systemic effects of immunotherapy, which could help to overcome resistance to immune checkpoint inhibitors (24).

In the murine model of metastatic melanoma and renal cancer, stereotactic radiotherapy combined with anti-PD-1 treatment showed a complete regression of the irradiated tumor, and the volume of distant metastatic tumors was significantly reduced (25). A patient who suffered metastatic renal cell carcinoma (RCC) achieved a systemic complete response in only 2.2 months of concurrent treatment combined with SBRT and pembrolizumab (26), which indicated that the combination of radiotherapy and immunotherapy may be a promising method to improve anti-tumor response. In a secondary analysis of the Phase I KEYNOTE-001 study, among the patients who received pembrolizumab, PFS and OS were significantly longer in those who received prior radiation therapy than those who did not (27). To the best of our knowledge, the PEMBRO-RT study is the first randomized trial to assess the synergistic effect of SBRT combined with PD-1 blockade in patients with metastatic NSCLC, overall response rate (ORR) increased from 18% in the control arm to 36% in the experimental arm (28). Clinic trials are ongoing to evaluate the optimal radiation dose, timing, and sequencing of radiation therapy and immunotherapy.

The abscopal effect refers to the clinical phenomenon whereby radiotherapy at the irradiated field may lead to regression of tumors in the non-irradiated area (29). Long-term anti-tumor immunity is very rarely observed by radiotherapy alone. Hence, blocking the PD-1/PD-L1 axis could alleviate the immune limitations of RT in the current era of cancer immunotherapy (30). A retrospective single-center study was conducted to evaluate abscopal effects during anti-PD-1 therapy and irradiation. Among 126 cancer patients who treated with PD-1 inhibitors and RT, 67 (53%) of 126 patients received concurrent combination therapy, and 24 (36%) of 67 patients met inclusion criteria, namely RT started within a month from the first and last dose of PD-1 inhibitors (pembrolizumab or nivolumab) and at least one metastasis outside the irradiation field. Abscopal effects were observed in 29% (7/24) patients (31). In addition, absolute lymphocyte count after radiation therapy may influence the occurrence of abscopal effects and prognosis in patients treated with RT and immunotherapy (32). Many ongoing clinical trials combining radiotherapy with checkpoint inhibitors are currently recruiting for different types of tumors (33) (e.g., NCT03557411, NCT03042156).

The combination of radiotherapy and immunotherapy is one of the current research focuses, several cases have confirmed the synergistic effect of immunotherapy and radiation. RT plus ICIs can relieve the intratumoral immunosuppression, increase tumor immunogenicity and tumor antigens release and presentation, eventually induce systemic tumor control (34). Programmed death ligand-1 (PD-L1) expression and mismatch repair deficiency (or microsatellite instability high) are FDA-approved biomarkers that predict response to ICIs in solid tumors treatment (35). Recent studies showed the expression of PD-L1 in thyroid cancer ranging between 23% and 87% (36). Increasing PD-L1 expression in PTC patients is associated with higher a risk of recurrence and shorter DFS, and may serve as a prognostic marker (5). To our knowledge, higher levels of PD-L1 expression in tumor tissues were associated with response rates and better survival in some ICIs clinical trials.

Furthermore, biological immunomodulators can improve the clinical response to anti-cancer treatment. cytokines such as GM-CSF, TNF-α, IFN-α, IL-2, IL-15, IL-12, and radiotherapy have synergistic effects (37). GM-CSF is an important cytokine for the regulation of anti-tumor immune response, which participates in the activation of innate and adaptive immunity. Preclinical data showed that GM-CSF in combination with RT can boost the abscopal effect (38). A proof-of-principle study suggested that GM-CSF combined with local radiotherapy lead to abscopal effects in some patients with metastatic solid tumors, resulting in an overall responses rate of 26.8% (11 patients out of 41) (39). Similarly, animal experiments showed that GM-CSF in combination with ICIs can improve antigen presentation and recruit T cells to infiltrate the tumor microenvironment, and thereby enhancing the effectiveness of PD-1/PD-L1 inhibitors (40, 41). A randomized clinical trial reported that in patients with unresectable stage III or IV melanoma, sargramostim plus ipilimumab had longer OS and lower toxicity compared to ipilimumab alone, but PFS had no difference (42).

To the best of our knowledge, there was just one case report of a PD-L1-negative patient with refractory metastatic esophageal squamous cell carcinoma who experienced significant systemic effect after receiving triple therapy comprising of radiotherapy, a PD-1 inhibitor, and GM-CSF, which indicated that radio-sensitization of immunotherapy might be involved in the underlying mechanism (43). Herein, we reported a patient with recurrent Hürthle cell carcinoma who underwent re-operation, radiotherapy, and targeted therapy, but the metastatic lesions continued to progress. This recurrent HCC case did not benefit from sorafenib. Given that the disease progressed after multi-line therapies, our team recommended immunotherapy for the patient. Numerous cases have shown that combined radiotherapy and GM-CSF could enhance systemic immune response, which is a promising therapy for both primary and distant metastases tumors. Although combination therapy can improve the curative effect, tolerance and adverse reactions should be monitored. Another case report innovatively used triple-combination therapy in esophageal squamous cell carcinoma (ESCC) and obtained short-term benefits, but the patient died of severe pneumonia (43). A Phase I trial of cemiplimab, radiotherapy, cyclophosphamide, and GM-CSF in patients with recurrent or metastatic head and neck squamous cell carcinoma, did not demonstrate higher efficacy than other PD-1 inhibitor monotherapies but showed tolerability of the regimen, and the most common treatment-emergent adverse events (TEAEs) included fatigue (40%), constipation (26.7%), asthenia, dyspnea, maculopapular rash, and pneumonia (20% each) (44). The SWORD trial reported the preliminary results of feasibility and safety of the triple combination of a PD-1/PD-L1 inhibitor, SBRT and GM-CSF in advanced solid tumors (45).

HCC led to a worse prognosis in cases with widely invasive tumors, male patients, older age, more than four foci of angioinvasion, tumor larger than 4 cm, and/or TNM stage III-IV (46). According to the recent American Thyroid Association (ATA) guidelines and NCCN Thyroid Cancer guidelines, for recurrent or metastatic HCC, treatment options include radioiodine, radiofrequency ablation, ethanol ablation, external beam radiotherapy, and systemic therapy (3). Nonetheless, options such as tyrosine kinase inhibitors (levantinib or sorafenib) show benefits in PFS, but not in OS. Moreover, a case report of a patient with progressive and metastatic HCC, found that recurrent HCC cases did not benefit from panitumumab and the criterion of RAS wild-type status seems to be insufficient for these recurrent thyroid cancer cases (47). Therefore, this case report may provide a possible treatment method for patients with advanced thyroid Hürthle cell carcinoma.

## Conclusion

This case report showed the efficacy of immunotherapy combined with distant radiotherapy and GM-CSF in a patient with thyroid Hürthle cell carcinoma. HCCs are poorly avid to radioiodine and poorly responsive to chemotherapy and radiation. A greater understanding of molecular pathogenesis and epigenomics will help to diagnose and manage thyroid cancers. Thyroid cancer is an immune-related tumor, and immunotherapy is a promising choice. ICIs are emerging as possible novel therapeutics for advanced thyroid carcinomas. The ongoing preclinical and clinical trials may prove the efficacy of immune monotherapy or combined with chemotherapy, radiotherapy, or targeted therapy.

## Data Availability Statement

The original contributions proposed in the study are included in the article/**Supplementary Material**. Further inquiries can be directed to the corresponding authors.

## Ethics Statement

The studies involving human participants were reviewed and approved by Human and Research Ethics committees of the Renmin Hospital of Wuhan University. The patients/participants provided their written informed consent to participate in this study. Written informed consent was obtained from the individual(s) for the publication of any potentially identifiable images or data included in this article.

## Author Contributions

HH collected data and wrote the manuscript. TX provided figures and pathology review and reviewed the manuscript. GJ and PL reviewed the manuscript. XL and QS, conception, organization, execution of the manuscript and review and critique of the manuscript. All authors contributed to the article and approved the submitted version.

## Funding

This research was supported by grants No.2014CFB394, 2019CFB721 from the Natural Science Foundation of Hubei Province (CN) and No.WJ2017M027 from Health and Family Planning Commission of Hubei Province (CN), No.Y-HS202101-0079 from Cisco hausen Cancer Research Foundation.

## Conflict of Interest

The authors declare that the research was conducted in the absence of any commercial or financial relationships that could be construed as a potential conflict of interest.

## Publisher’s Note

All claims expressed in this article are solely those of the authors and do not necessarily represent those of their affiliated organizations, or those of the publisher, the editors and the reviewers. Any product that may be evaluated in this article, or claim that may be made by its manufacturer, is not guaranteed or endorsed by the publisher.

## References

[1] Cameselle-Teijeiro JM Sobrinho-Simoes M. New WHO Classification of Thyroid Tumors: A Pragmatic Categorization of Thyroid Gland Neoplasms. Endocrinol Diabetes Nutr (Engl Ed) (2018) 65(3):133–5. doi: 10.1016/j.endinu.2017.11.012 29396216

[2] Haugen BR Alexander EK Bible KC Doherty GM Mandel SJ Nikiforov YE. 2015 American Thyroid Association Management Guidelines for Adult Patients With Thyroid Nodules and Differentiated Thyroid Cancer: The American Thyroid Association Guidelines Task Force on Thyroid Nodules and Differentiated Thyroid Cancer. Thyroid (2016) 26(1):1–133. doi: 10.1089/thy.2015.0020 26462967 PMC4739132

[3] Canberk S Lima AR Pinto M Soares P Maximo V. Epigenomics in Hurthle Cell Neoplasms: Filling in the Gaps Towards Clinical Application. Front Endocrinol (Lausanne) (2021) 12:674666. doi: 10.3389/fendo.2021.674666 34108939 PMC8181423

[4] Erickson LA Jin L Goellner JR Lohse C Pankratz VS Zukerberg LR. Pathologic Features, Proliferative Activity, and Cyclin D1 Expression in Hurthle Cell Neoplasms of the Thyroid. Modern Pathol (2000) 13(2):186–92. doi: 10.1038/modpathol.3880034 10697277

[5] Chowdhury S Veyhl J Jessa F Polyakova O Alenzi A MacMillan C. Programmed Death-Ligand 1 Overexpression Is a Prognostic Marker for Aggressive Papillary Thyroid Cancer and Its Variants. Oncotarget (2016) 7(22):32318–28. doi: 10.18632/oncotarget.8698 PMC5078015 27086918

[6] Kushchayeva Y Duh QY Kebebew E Clark OH. Prognostic Indications for Hurthle Cell Cancer. World J Surg (2004) 28(12):1266–70. doi: 10.1007/s00268-004-7602-2 15517492

[7] Laha D Nilubol N Boufraqech M. New Therapies for Advanced Thyroid Cancer. Front Endocrinol (2020) 11:82. doi: 10.3389/fendo.2020.00082 PMC7257776 32528402

[8] French JD. Immunotherapy for Advanced Thyroid Cancers - Rationale, Current Advances and Future Strategies. Nat Rev Endocrinol (2020) 16(11):629–41. doi: 10.1038/s41574-020-0398-9 32839578

[9] Moretti S Menicali E Nucci N Guzzetti M Morelli S Puxeddu E. THERAPY OF ENDOCRINE DISEASE Immunotherapy of Advanced Thyroid Cancer: From Bench to Bedside. Eur J Endocrinol (2020) 183(2):R41–55. doi: 10.1530/Eje-20-0283 32449696

[10] Galon J Bruni D. Approaches to Treat Immune Hot, Altered and Cold Tumours With Combination Immunotherapies. Nat Rev Drug Discovery (2019) 18(3):197–218. doi: 10.1038/s41573-018-0007-y 30610226

[11] Mills SC Haq M Smellie WJ Harmer C. Hürthle Cell Carcinoma of the Thyroid: Retrospective Review of 62 Patients Treated at the Royal Marsden Hospital Between 1946 and 2003. Eur J Surg Oncol J Eur Soc Surg Oncol Br Assoc Surg Oncol (2009) 35(3):230–4. doi: 10.1016/j.ejso.2008.06.007 18722077

[12] Besic N Vidergar-Kralj B Frkovic-Grazio S Movrin-Stanovnik T Auersperg M. The Role of Radioactive Iodine in the Treatment of Hurthle Cell Carcinoma of the Thyroid. Thyroid (2003) 13(6):577–84. doi: 10.1089/105072503322238845 12930602

[13] Oluic B Paunovic I Loncar Z Djukic V Diklic A Jovanovic M. Survival and Prognostic Factors for Survival, Cancer Specific Survival and Disease Free Interval in 239 Patients With Hurthle Cell Carcinoma: A Single Center Experience. BMC Cancer (2017) 17(1):371. doi: 10.1186/s12885-017-3370-x 28545571 PMC5445517

[14] Guerrero MA Suh I Vriens MR Shen WT Gosnell J Kebebew E. Age and Tumor Size Predicts Lymph Node Involvement in Hurthle Cell Carcinoma. J Cancer (2010) 1:23–6. doi: 10.7150/jca.1.23 PMC2931345 20842220

[15] Wenter V Albert NL Unterrainer M Ahmaddy F Ilhan H Jellinek A. Clinical Impact of Follicular Oncocytic (Hurthle Cell) Carcinoma in Comparison With Corresponding Classical Follicular Thyroid Carcinoma. Eur J Nucl Med Mol Imaging (2021) 48(2):449–60. doi: 10.1007/s00259-020-04952-2 PMC7835150 32683470

[16] Nabhan F Dedhia PH Ringel MD. Thyroid Cancer, Recent Advances in Diagnosis and Therapy. Int J Cancer (2021) 149(5):984–92. doi: 10.1002/ijc.33690 34013533

[17] Mehnert JM Varga A Brose MS Aggarwal RR Lin CC Prawira A. Safety and Antitumor Activity of the Anti-PD-1 Antibody Pembrolizumab in Patients With Advanced, PD-L1-Positive Papillary or Follicular Thyroid Cancer. BMC Cancer (2019) 19(1):196. doi: 10.1186/s12885-019-5380-3 30832606 PMC6399859

[18] Bagchi S Yuan R Engleman EG. Immune Checkpoint Inhibitors for the Treatment of Cancer: Clinical Impact and Mechanisms of Response and Resistance. Annu Rev Pathol (2021) 16:223–49. doi: 10.1146/annurev-pathol-042020-042741 33197221

[19] Mo DC Huang JF Luo PH Huang SX Wang HL. The Efficacy and Safety of Combination Therapy With Immune Checkpoint Inhibitors in Non-Small Cell Lung Cancer: A Meta-Analysis. Int Immunopharmacol (2021) 96:107594. doi: 10.1016/j.intimp.2021.107594 33798808

[20] Eriksson D Stigbrand T. Radiation-Induced Cell Death Mechanisms. Tumour Biol (2010) 31(4):363–72. doi: 10.1007/s13277-010-0042-8 20490962

[21] Golden EB Marciscano AE Formenti SC. Radiation Therapy and the *In Situ* Vaccination Approach. Int J Radiat Oncol Biol Phys (2020) 108(4):891–8. doi: 10.1016/j.ijrobp.2020.08.023 32800803

[22] Wilkins AC Patin EC Harrington KJ Melcher AA. The Immunological Consequences of Radiation-Induced DNA Damage. J Pathol (2019) 247(5):606–14. doi: 10.1002/path.5232 30632153

[23] Sharabi AB Lim M DeWeese TL Drake CG. Radiation and Checkpoint Blockade Immunotherapy: Radiosensitisation and Potential Mechanisms of Synergy. Lancet Oncol (2015) 16(13):e498–509. doi: 10.1016/S1470-2045(15)00007-8 26433823

[24] Bang A Schoenfeld JD. Immunotherapy and Radiotherapy for Metastatic Cancers. Ann Palliat Med (2019) 8(3):312–25. doi: 10.21037/apm.2018.07.10 30180743

[25] Park SS Dong H Liu X Harrington SM Krco CJ Grams MP. PD-1 Restrains Radiotherapy-Induced Abscopal Effect. Cancer Immunol Res (2015) 3(6):610–9. doi: 10.1158/2326-6066.Cir-14-0138 PMC4827718 25701325

[26] Xie GZ Gu D Zhang LF Chen SJ Wu DH. A Rapid and Systemic Complete Response to Stereotactic Body Radiation Therapy and Pembrolizumab in a Patient With Metastatic Renal Cell Carcinoma. Cancer Biol Ther (2017) 18(8):547–51. doi: 10.1080/15384047.2017.1345389 PMC5652971 28665741

[27] Shaverdian N Lisberg AE Bornazyan K Veruttipong D Goldman JW Formenti SC. Previous Radiotherapy and the Clinical Activity and Toxicity of Pembrolizumab in the Treatment of Non-Small-Cell Lung Cancer: A Secondary Analysis of the KEYNOTE-001 Phase 1 Trial. Lancet Oncol (2017) 18(7):895–903. doi: 10.1016/S1470-2045(17)30380-7 28551359 PMC5538772

[28] Theelen W Peulen HMU Lalezari F van der Noort V de Vries JF Aerts J. Effect of Pembrolizumab After Stereotactic Body Radiotherapy vs Pembrolizumab Alone on Tumor Response in Patients With Advanced Non-Small Cell Lung Cancer: Results of the PEMBRO-RT Phase 2 Randomized Clinical Trial. JAMA Oncol (2019) 5(9):1276–82. doi: 10.1001/jamaoncol.2019.1478 PMC6624814 31294749

[29] Mole RH. Whole Body Irradiation; Radiobiology or Medicine? Br J Radiol (1953) 26(305):234–41. doi: 10.1259/0007-1285-26-305-234 13042090

[30] Dovedi SJ Adlard AL Lipowska-Bhalla G McKenna C Jones S Cheadle EJ. Acquired Resistance to Fractionated Radiotherapy Can be Overcome by Concurrent PD-L1 Blockade. Cancer Res (2014) 74(19):5458–68. doi: 10.1158/0008-5472.Can-14-1258 25274032

[31] Trommer M Yeo SY Persigehl T Bunck A Grüll H Schlaak M. Abscopal Effects in Radio-Immunotherapy-Response Analysis of Metastatic Cancer Patients With Progressive Disease Under Anti-PD-1 Immune Checkpoint Inhibition. Front Pharmacol (2019) 10:511. doi: 10.3389/fphar.2019.00511 31156434 PMC6530339

[32] Chen D Verma V Patel RR Barsoumian HB Cortez MA Welsh JW. Absolute Lymphocyte Count Predicts Abscopal Responses and Outcomes in Patients Receiving Combined Immunotherapy and Radiation Therapy: Analysis of 3 Phase 1/2 Trials. Int J Radiat Oncol Biol Phys (2020) 108(1):196–203. doi: 10.1016/j.ijrobp.2020.01.032 32036004

[33] Bockel S Durand B Deutsch E. Combining Radiation Therapy and Cancer Immune Therapies: From Preclinical Findings to Clinical Applications. Cancer Radiother (2018) 22(6-7):567–80. doi: 10.1016/j.canrad.2018.07.136 30197026

[34] Kong Y Ma Y Zhao X Pan J Xu Z Zhang L. Optimizing the Treatment Schedule of Radiotherapy Combined With Anti-PD-1/PD-L1 Immunotherapy in Metastatic Cancers. Front Oncol (2021) 11:638873. doi: 10.3389/fonc.2021.638873 33859942 PMC8042160

[35] Shum B Larkin J Turajlic S. Predictive Biomarkers for Response to Immune Checkpoint Inhibition. Semin Cancer Biol (2021) S1044-579X(21)00097-3. doi: 10.1016/j.semcancer.2021.03.036 33819567

[36] Ahn S Kim TH Kim SW Ki CS Jang HW Kim JS. Comprehensive Screening for PD-L1 Expression in Thyroid Cancer. Endocr Relat Cancer (2017) 24(2):97–106. doi: 10.1530/ERC-16-0421 28093480

[37] Palata O Hradilova Podzimkova N Nedvedova E Umprecht A Sadilkova L Palova Jelinkova L. Radiotherapy in Combination With Cytokine Treatment. Front Oncol (2019) 9:367. doi: 10.3389/fonc.2019.00367 31179236 PMC6538686

[38] Demaria S Ng B Devitt ML Babb JS Kawashima N Liebes L. Ionizing Radiation Inhibition of Distant Untreated Tumors (Abscopal Effect) Is Immune Mediated. Int J Radiat Oncol Biol Phys (2004) 58(3):862–70. doi: 10.1016/j.ijrobp.2003.09.012 14967443

[39] Golden EB Chhabra A Chachoua A Adams S Donach M Fenton-Kerimian M. Local Radiotherapy and Granulocyte-Macrophage Colony-Stimulating Factor to Generate Abscopal Responses in Patients With Metastatic Solid Tumours: A Proof-of-Principle Trial. Lancet Oncol (2015) 16(7):795–803. doi: 10.1016/s1470-2045(15)00054-6 26095785

[40] Everly JJ Lonial S. Immunomodulatory Effects of Human Recombinant Granulocyte-Macrophage Colony-Stimulating Factor (rhuGM-CSF): Evidence of Antitumour Activity. Expert Opin Biol Ther (2005) 5(3):293–311. doi: 10.1517/14712598.5.3.293 15833068

[41] Gurbatri CR Lia I Vincent R Coker C Castro S Treuting PM. Engineered Probiotics for Local Tumor Delivery of Checkpoint Blockade Nanobodies. Sci Transl Med (2020) 12(530):eaax0876. doi: 10.1126/scitranslmed.aax0876 32051224 PMC7685004

[42] Hodi FS Lee S McDermott DF Rao UN Butterfield LH Tarhini AA. Ipilimumab Plus Sargramostim vs Ipilimumab Alone for Treatment of Metastatic Melanoma: A Randomized Clinical Trial. JAMA (2014) 312(17):1744–53. doi: 10.1001/jama.2014.13943 PMC4336189 25369488

[43] Zhao X Kong Y Zhang L. Anti-PD-1 Immunotherapy Combined With Stereotactic Body Radiation Therapy and GM-CSF as Salvage Therapy in a PD-L1-Negative Patient With Refractory Metastatic Esophageal Squamous Cell Carcinoma: A Case Report and Literature Review. Front Oncol (2020) 10:1625. doi: 10.3389/fonc.2020.01625 33014817 PMC7493754

[44] Babiker H Brana I Mahadevan D Owonikoko T Calvo E Rischin D. Phase I Trial of Cemiplimab, Radiotherapy, Cyclophosphamide, and Granulocyte Macrophage Colony-Stimulating Factor in Patients With Recurrent or Metastatic Head and Neck Squamous Cell Carcinoma. Oncologist (2021) 26(9):e1508–e13. doi: 10.1002/onco.13810 PMC8417861 33942954

[45] Ni J Zhou Y Wu L Ai X Dong X Chu Q. Sintilimab, Stereotactic Body Radiotherapy and Granulocyte-Macrophage Colony Stimulating Factor as Second-Line Therapy for Advanced Non-Small Cell Lung Cancer: Safety Run-in Results of a Multicenter, Single-Arm, Phase II Trial. Radiat Oncol (London England) (2021) 16(1):177. doi: 10.1186/s13014-021-01905-3 PMC8444553 34526044

[46] Kure S Ohashi R. Thyroid Hurthle Cell Carcinoma: Clinical, Pathological, and Molecular Features. Cancers (Basel) (2020) 13(1):26. doi: 10.3390/cancers13010026 PMC7793513 33374707

[47] Aydemirli MD Corver W Beuk R Roepman P Solleveld-Westerink N van Wezel T. Targeted Treatment Options of Recurrent Radioactive Iodine Refractory Hurthle Cell Cancer. Cancers (Basel) (2019) 11(8):1185. doi: 10.3390/cancers11081185 PMC6721552 31443247

