# Supplementary figures and images for: Anti-PD-1 Immunotherapy Combined With Stereotactic Body Radiation Therapy and GM-CSF as Salvage Therapy in a PD-L1-Positive Patient With Refractory Metastatic Thyroid Hürthle Cell Carcinoma: A Case Report and Literature Review
Source: Front Oncol. 2021 Nov 23;11:782646. doi: 10.3389/fonc.2021.782646 (PMC8650693; doi:10.3389/fonc.2021.782646)

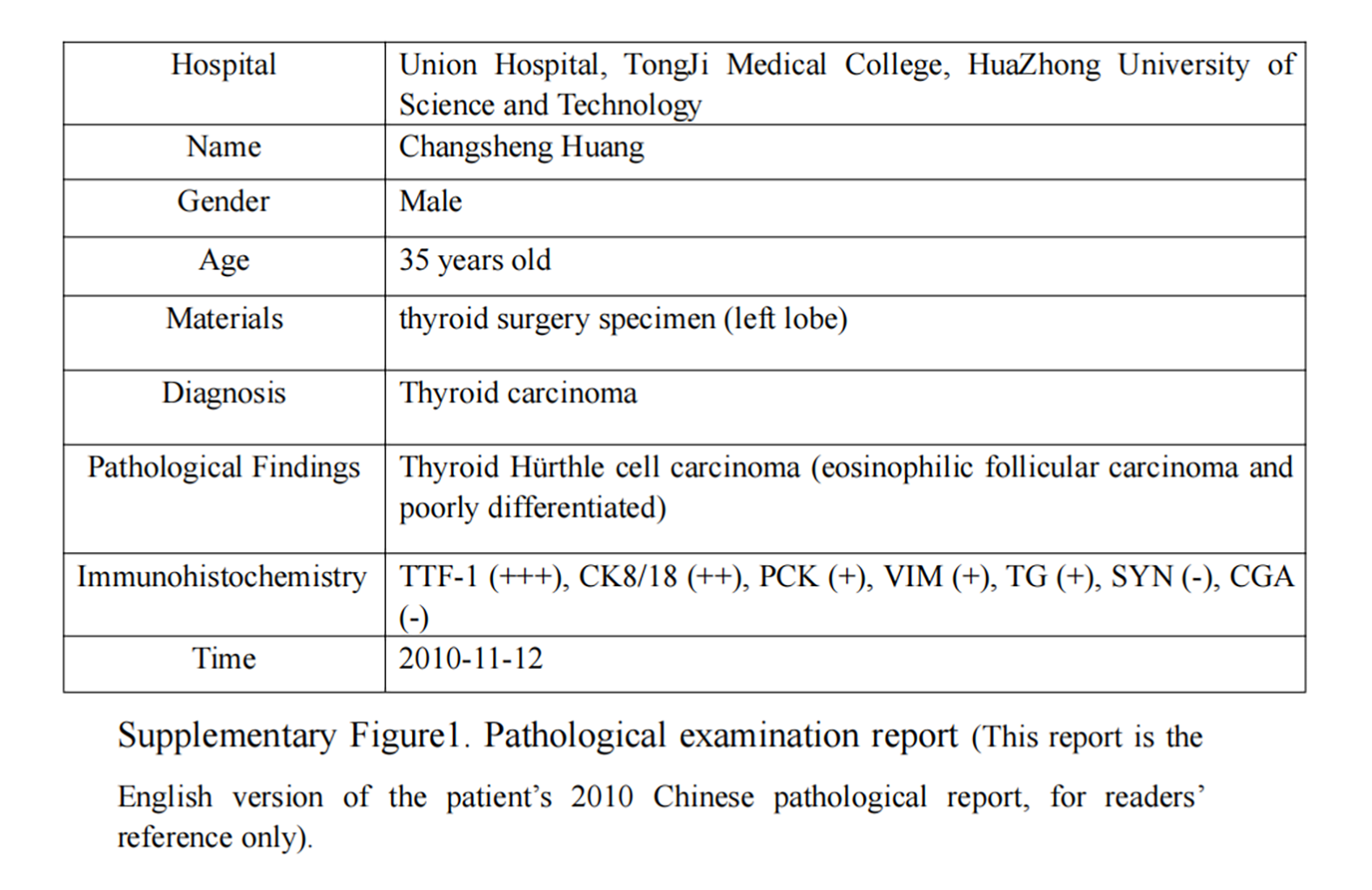

Supplement: Supplementary file 1 [file Image_1.tif]

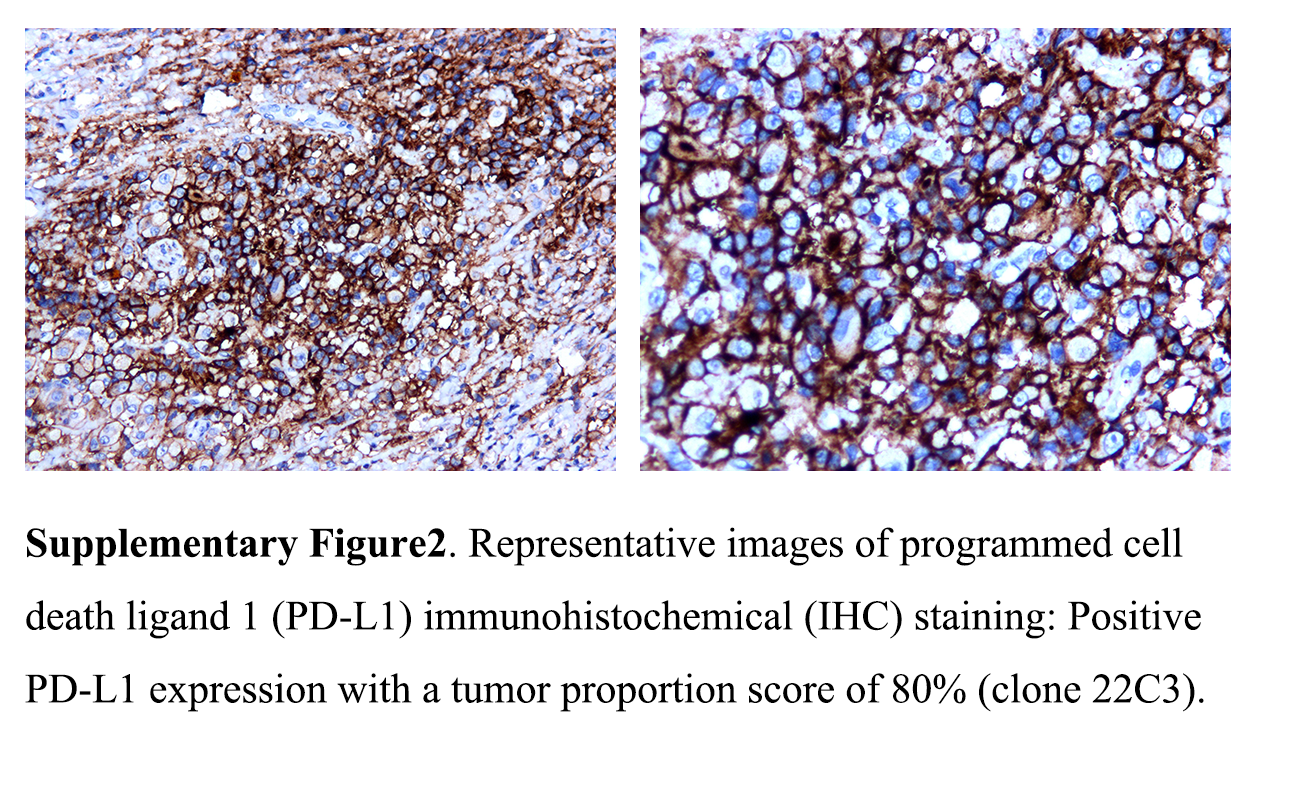

Supplement: Supplementary file 2 [file Image_2.tif]

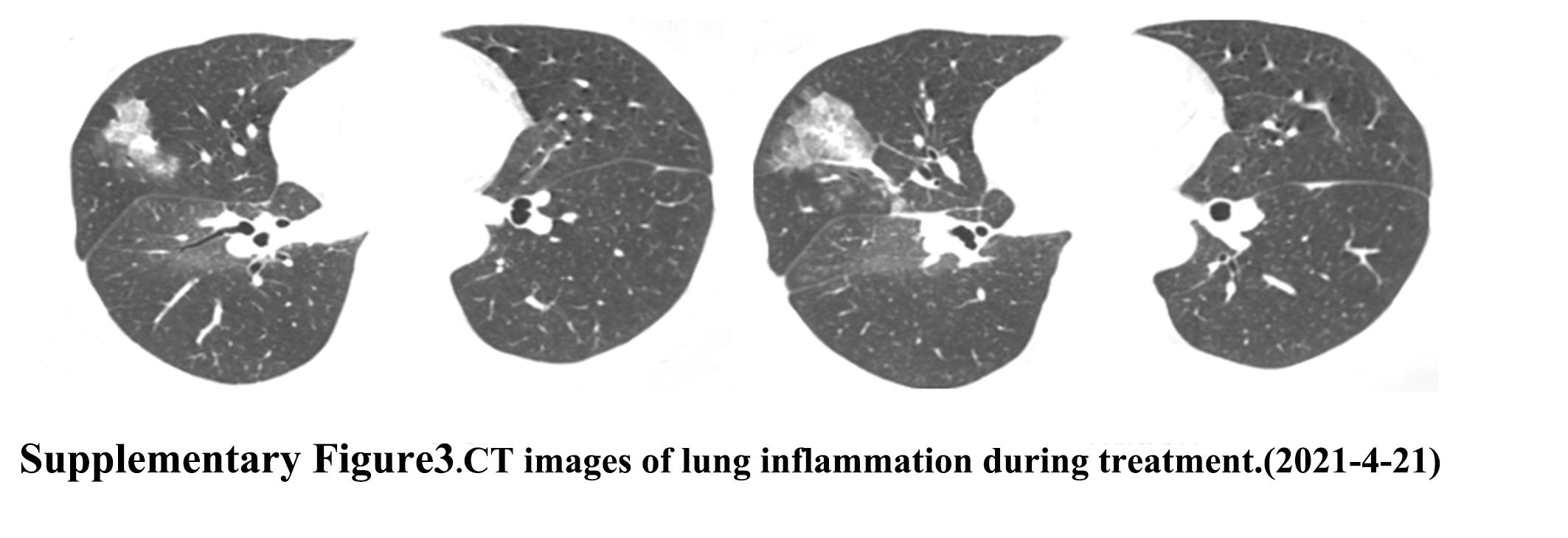

Supplement: Supplementary file 3 [file Image_3.tif]
